# Obesity-Related Phenotype of Heart Failure With Preserved Ejection Fraction: A Comprehensive Review

**DOI:** 10.7759/cureus.81512

**Published:** 2025-03-31

**Authors:** Taha El Hadj Othmane, Omar El Hadj Othmane, Hisham Nizar

**Affiliations:** 1 Cardiology, Heart Failure and Hypertension, OMC Healthcare, London, GBR; 2 Sixth Form, Harris Academy South Norwood, London, GBR; 3 Acute Medicine, Clinical Pharmacology, Croydon University Hospital, London, GBR

**Keywords:** cardiac remodeling, heart failure with preserved ejection fraction, metabolic dysregulation, obesity, obesity-related comorbidities, obesity-related heart failure

## Abstract

Heart failure with preserved ejection fraction (HFpEF) is a complex clinical syndrome with an obesity-related phenotype gaining prominence amid the global obesity epidemic. This review explores the distinct pathophysiological mechanisms, diagnostic challenges, and management strategies associated with obesity-induced HFpEF. Obesity contributes to HFpEF through several key mechanisms, including increased blood volume, myocardial hypertrophy and fibrosis, systemic inflammation, and metabolic dysregulation. These factors collectively exacerbate diastolic dysfunction and elevate left ventricular filling pressures, hallmark features of HFpEF. Diagnosing HFpEF in obese patients is particularly challenging due to overlapping comorbidities such as hypertension and diabetes, as well as the reduced reliability of traditional biomarkers such as N-terminal pro-B-type natriuretic peptide. Advanced imaging techniques are crucial in assessing diastolic dysfunction and myocardial remodeling. Managing obesity-related HFpEF requires a comprehensive approach. Lifestyle modifications, including weight loss and exercise, form the cornerstone of treatment, complemented by pharmacological therapies such as sodium-glucose cotransporter 2 inhibitors and mineralocorticoid receptor antagonists. Optimizing comorbidity management is essential, while emerging therapies targeting inflammation, fibrosis, and metabolic dysfunction, alongside precision medicine approaches, offer promising future advancements. This review underscores the need for inclusive clinical trials and personalized treatment strategies to improve outcomes in obesity-related HFpEF. A deeper understanding of this phenotype is crucial for developing targeted interventions that enhance patient care and quality of life. Integrating these insights into clinical practice can help optimize diagnostic accuracy, refine therapeutic approaches, and guide risk stratification for better patient management.

## Introduction and background

Heart failure with preserved ejection fraction (HFpEF) is a prevalent and complex syndrome marked by heart failure symptoms despite a normal left ventricular ejection fraction [1]. Unlike heart failure with reduced ejection fraction, HFpEF has a more intricate pathophysiology and a broader range of phenotypes, further complicating diagnosis and treatment [2]. Among these phenotypes, the obesity-related HFpEF subtype has garnered increasing attention due to the global rise in obesity rates and its distinct clinical and pathophysiological features [3]. In contrast to other HFpEF phenotypes, obesity-related HFpEF is driven by chronic volume overload, low natriuretic peptide levels, and metabolic dysregulation, which alter conventional diagnostic and treatment approaches. Furthermore, obesity itself modifies HFpEF presentation by reducing the reliability of traditional biomarkers and influencing cardiac remodeling patterns. As obesity rates continue to rise globally, recognizing and addressing this distinct HFpEF subtype is crucial for improving patient outcomes.

Obesity, characterized by excessive body fat accumulation, is a well-established risk factor for cardiovascular diseases, including coronary artery disease, hypertension, and heart failure [4]. The increasing prevalence of obesity has corresponded with a rise in HFpEF cases [5]. Research shows that a significant proportion of HFpEF patients are obese, with obesity playing a crucial role in the development and progression of the syndrome through multiple mechanisms [6,7].

The pathophysiology of obesity-related HFpEF involves a complex interaction of hemodynamic, structural, metabolic, and inflammatory changes [8]. Obesity is associated with increased blood volume, which can elevate cardiac output in some individuals. However, this relationship is multifaceted and varies depending on factors such as the severity of obesity and ventricular adaptation. These changes, together with increased left ventricular filling pressures, contribute to diastolic dysfunction, a key feature of HFpEF [9]. Additionally, myocardial hypertrophy and fibrosis, driven by metabolic and inflammatory processes, increase cardiac stiffness and impair diastolic function [10]. Together, these alterations distinguish the obesity-related HFpEF phenotype from other forms of HFpEF.

Diagnosing HFpEF in obese individuals presents unique challenges. Traditional biomarkers such as natriuretic peptides, commonly used to diagnose heart failure, often yield lower levels in obese patients, complicating diagnosis [8]. Moreover, the frequent coexistence of obesity with other conditions such as hypertension, diabetes, and obstructive sleep apnea further complicates the clinical picture, making it hard to attribute symptoms solely to HFpEF [11,12].

Managing obesity-related HFpEF requires a multifaceted approach that addresses both the underlying obesity and associated cardiac dysfunction. Lifestyle interventions, including weight loss through diet and exercise, have shown benefits in improving cardiac function and symptoms in obese HFpEF patients [13,14]. However, pharmacological treatments have limitations, highlighting the need for novel therapies targeting this phenotype [8].

This review provides an overview of the obesity-related HFpEF phenotype, focusing on its pathophysiology, diagnostic challenges, and management strategies. By summarizing current knowledge and identifying research gaps, it aims to inform clinical practice and guide future studies into this growing form of heart failure. Understanding the unique characteristics of obesity-related HFpEF is essential for developing tailored therapeutic approaches and improving patient outcomes.

## Review

Pathophysiology of obesity-related HFpEF

Obesity-related HFpEF arises from a complex interplay of hemodynamic changes, myocardial remodeling, inflammation, and metabolic dysregulation. Excess body weight drives cardiovascular alterations that contribute to diastolic dysfunction, impaired ventricular relaxation, and, ultimately, to HFpEF [8,9]. These changes compromise left ventricular filling, leading to elevated filling pressures and heart failure symptoms despite a preserved ejection fraction [10,11].

Hemodynamic Changes

A hallmark of obesity-related HFpEF is altered hemodynamics driven by increased blood volume and vascular resistance. Obesity can elevate cardiac output to meet the heightened metabolic demands of excess adipose tissue, though this effect varies based on obesity severity and individual cardiac adaptation [15]. The resulting volume overload raises left ventricular filling pressures, a key contributor to diastolic dysfunction.

Excess adiposity increases vascular resistance, particularly in the peripheral and splanchnic circulations, prompting a compensatory rise in cardiac output [16]. Over time, chronic hypervolemia and excessive preload place added strain on the heart, leading to elevated left atrial pressure as the left ventricle struggles to accommodate the increased blood volume [15,17]. Impaired left ventricular relaxation further elevates filling pressures during early diastole, restricting proper ventricular filling and driving HFpEF symptoms [18].

Additionally, sympathetic overactivity in obesity heightens heart rate and contractility, exacerbating hemodynamic stress and accelerating the progression of diastolic dysfunction, a central feature of obesity-related HFpEF [19].

Myocardial Remodeling

Obesity induces myocardial remodeling, progressively impairing cardiac function. Structural and functional adaptations occur in response to mechanical stress from excess body mass and elevated hemodynamic load [20]. A key feature of obesity-related HFpEF is left ventricular hypertrophy, an adaptive response to chronic pressure and volume overload [21]. This hypertrophy, marked by an increase in myocyte size and myocardial wall thickening, initially compensates for increased workload but ultimately reduces myocardial compliance, impairing diastolic function [10,22,23].

Beyond hypertrophy, myocardial fibrosis plays a central role in obesity-related HFpEF [21]. Visceral adipose tissue secretes adipokines (e.g., leptin, adiponectin, resistin), growth factors, and inflammatory cytokines, which activate fibrotic pathways and promote extracellular matrix deposition [23,24]. The accumulation of collagen and other fibrotic proteins increases myocardial stiffness, further reducing ventricular compliance and worsening diastolic dysfunction [21].

The combined effects of hypertrophy and fibrosis reduce myocardial distensibility, limiting diastolic filling and elevating left ventricular filling pressures. This progressively impairs preload handling, exacerbating diastolic dysfunction and advancing HFpEF.

Inflammation and Metabolic Dysregulation

Inflammation and metabolic dysregulation are key drivers of obesity-related HFpEF. Visceral adipose tissue functions as an endocrine organ, releasing pro-inflammatory cytokines such as tumour necrosis factor-alpha (TNF-α), interleukin-6 (IL-6), and monocyte chemoattractant protein-1 [25]. These cytokines induce systemic inflammation, which worsens endothelial dysfunction, increases vascular stiffness, and accelerates atherosclerosis [26]. Chronic inflammation contributes to myocardial injury, endothelial damage, and fibrosis, all of which play a role in HFpEF development [27].

Inflammatory mediators also directly affect the myocardium, triggering diastolic dysfunction and fibrosis [28]. Myocardial inflammation disrupts cardiomyocyte function and activates signaling pathways that drive extracellular matrix remodeling, impairing ventricular relaxation and filling during diastole [28,29].

In addition to inflammation and metabolic dysregulation, insulin resistance and dyslipidemia further exacerbate myocardial dysfunction [30]. Insulin resistance, a hallmark of obesity, leads to hyperinsulinemia, which promotes collagen synthesis and pro-fibrotic pathways in cardiomyocytes [30,31]. Additionally, it impairs mitochondrial function and increases oxidative stress, accelerating myocardial injury and fibrosis [32].

Dyslipidemia, characterized by elevated low-density lipoprotein cholesterol and triglycerides, also contributes to cardiovascular dysfunction [33]. Myocardial lipid accumulation induces lipotoxicity, causing cardiomyocyte apoptosis, myocardial injury, and fibrosis [34]. Together, insulin resistance and dyslipidemia accelerate diastolic dysfunction, reinforcing the progression of HFpEF in obese individuals [33-35].

**Figure 1:**
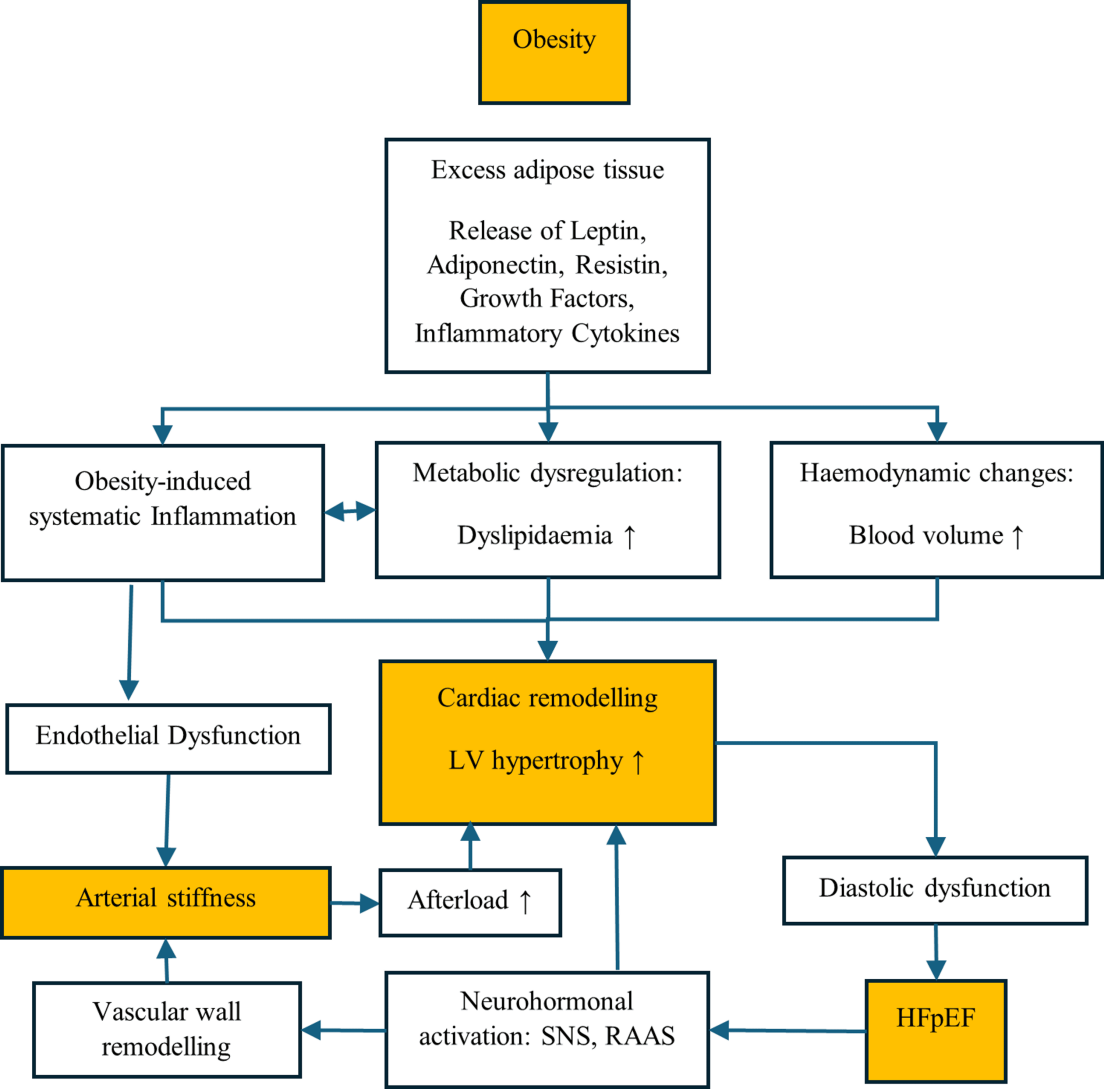
Pathophysiology of obesity-related HFpEF phenotype. HFpEF: heart failure with preserved ejection fraction; LV: left ventricular; LA: left atrial; SNS: sympathetic nervous system; RAAS: renin-angiotensin-aldosterone system This figure is the original creation of the authors.

In conclusion, obesity-related HFpEF results from a convergence of interrelated pathophysiological mechanisms (Figure 1). Hemodynamic changes, myocardial remodeling, systemic inflammation, and metabolic dysregulation contribute to diastolic dysfunction and impaired ventricular relaxation. While obesity-associated cardiac output elevations can raise left ventricular filling pressures, this effect varies with obesity severity and cardiac adaptation. Myocardial hypertrophy and fibrosis further compromise myocardial compliance, while inflammation and metabolic dysfunction perpetuate a vicious cycle that exacerbates HFpEF. Understanding these mechanisms is essential for developing targeted therapies that address the root causes of obesity-related HFpEF.

Diagnostic challenges of obesity-related HFpEF

Diagnosing obesity-related HFpEF is challenging due to overlapping comorbidities, the limited reliability of conventional biomarkers, and the complexity of imaging assessments required to evaluate diastolic function and myocardial remodeling. These factors make the differentiation of obesity-related HFpEF from other conditions with similar clinical presentations challenging. This emphasizes the need for specialized diagnostic strategies.

Overlap With Other Comorbidities

Obesity commonly coexists with hypertension, type 2 diabetes, and obstructive sleep apnea (OSA), all of which can independently contribute to HFpEF development or symptom exacerbation [35,36]. These overlapping conditions make it hard to attribute clinical findings solely to obesity [37].

Hypertension, one of the most prevalent comorbidities in obesity, is a well-established risk factor for HFpEF [37,38]. Elevated blood pressure can mask or mimic HFpEF pathophysiology by independently causing diastolic dysfunction and increasing left ventricular filling pressures [38]. Similarly, insulin resistance and type 2 diabetes promote myocardial fibrosis, endothelial dysfunction, and metabolic dysregulation, further complicating the differentiation between obesity-related HFpEF and other heart failure subtypes [31,32].

OSA, frequently seen in obesity, contributes to the progression of HFpEF through intermittent hypoxia, systemic inflammation, and increased sympathetic tone [39,40]. By aggravating diastolic dysfunction, OSA can worsen HFpEF symptoms [39]. Given these interconnected pathophysiological mechanisms, accurately diagnosing HFpEF in obese patients requires a comprehensive assessment that accounts for all contributing factors.

Role of Biomarkers

N-terminal pro-B-type natriuretic peptide (NT-proBNP) and B-type natriuretic peptide (BNP) are widely used to diagnose heart failure, as they rise in response to ventricular wall stress and fluid overload [41]. However, their reliability is reduced in obese patients [42]. Studies indicate that NT-proBNP and BNP levels tend to be lower in obesity, even in HFpEF, due to altered metabolic and hormonal regulation, leading to underestimation of heart failure severity and potential false-negative results [43].

This reduced biomarker sensitivity is attributed to several obesity-related factors, including increased blood volume, which dilutes natriuretic peptide concentrations, and adiposity-related changes in renal clearance, further lowering circulating levels [44,45]. Consequently, relying solely on NT-proBNP or BNP may be insufficient for diagnosing HFpEF in obese patients, necessitating adjusted thresholds or alternative biomarkers [46].

Emerging biomarkers of fibrosis (e.g., galectin-3) and inflammation (e.g., high-sensitivity C-reactive protein) may offer additional diagnostic value in obesity-related HFpEF [47]. However, further research is needed to establish their clinical utility and diagnostic accuracy.

Imaging Modalities

Given the limitations of biomarkers in obese patients, advanced imaging techniques are essential for diagnosing obesity-related HFpEF. Echocardiography and cardiac MRI are crucial in assessing diastolic function and myocardial remodeling [48].

Echocardiography is a widely accessible, non-invasive modality that evaluates left ventricular diastolic function, chamber dimensions, and wall thickness. Key indices, including the E/A ratio, E/e' ratio, and left atrial volume, provide valuable insights into the diastolic function and filling pressures [49]. However, in obese patients, increased chest wall thickness can interfere with sound wave propagation, potentially reducing image quality and diagnostic accuracy when assessing left ventricular filling pressures [50].

Cardiac MRI offers higher resolution and precise evaluation of myocardial structure and function. It effectively assesses left ventricular hypertrophy, myocardial fibrosis, and ventricular stiffness, hallmarks of obesity-related HFpEF [51]. Additionally, MRI provides a comprehensive assessment of systolic and diastolic function, myocardial edema, and inflammation, helping to differentiate HFpEF from other conditions [52]. Although highly effective, using cardiac MRI is restricted by accessibility, cost, and the need for specialized equipment and expertise.

When combined, echocardiography and cardiac MRI enhance diagnostic accuracy, helping to overcome the challenges posed by comorbidities, biomarker limitations, and other complexities in obese HFpEF patients [48]. Table 1 summarizes the key diagnostic challenges and potential solutions.

**Table 1:** Key diagnostic challenges and their potential solutions in obesity-related HFpEF. HFpEF: heart failure with preserved ejection fraction; OSA: obstructive sleep apnea; CPAP: continuous positive airway pressure; NT-proBNP: N-terminal pro-B-type natriuretic peptide; BNP: B-type natriuretic peptide; hs-CRP: high-sensitivity C-reactive protein

Challenge	Explanation	Potential solutions	References
Overlap with Comorbidities	Obesity often coexists with conditions such as hypertension, type 2 diabetes, and OSA, which share symptoms with HFpEF and complicate diagnosis	Comprehensive clinical assessments, detailed medical history, and considering all possible contributing factors	[35-37]
Hypertension	Elevated blood pressure can cause diastolic dysfunction independently, making it hard to distinguish from HFpEF	Careful evaluation of blood pressure control and using imaging techniques to assess specific markers of HFpEF	[37,38]
Type 2 diabetes and insulin resistance	These conditions can lead to myocardial fibrosis and endothelial dysfunction, which mimic HFpEF symptoms	Integrating metabolic profiling and assessing for diabetic complications alongside heart failure evaluations	[31,32]
OSA	OSA contributes to HFpEF through intermittent hypoxia and increased sympathetic activity, worsening diastolic dysfunction	Screening for and treating OSA using polysomnography and CPAP therapy	[39,40]
Reduced sensitivity of biomarkers	NT-proBNP and BNP levels are lower in obese patients, which can lead to false negatives in diagnosing HFpEF	Using adjusted thresholds for these biomarkers or integrating alternative markers such as galectin-3 or hs-CRP	[41-43]
Limitations of NT-proBNP and BNP	The dilution effect from increased blood volume and altered clearance mechanisms in obesity reduce the effectiveness of these biomarkers	Employing a multi-marker strategy and considering clinical context when interpreting NT-proBNP and BNP levels	[44-46]
Emerging biomarkers	Newer markers such as galectin-3 and hs-CRP may provide additional information, but are not yet fully validated for HFpEF diagnosis	Ongoing research and clinical trials to establish the reliability and cut-off values for these emerging biomarkers	[47]
Imaging limitations in obesity	Echocardiography can be less accurate in obese patients due to poor image quality caused by increased chest wall thickness	Using advanced echocardiographic techniques such as tissue Doppler or switching to cardiac MRI for better resolution	[48-50]
Accessibility of cardiac MRI	While cardiac MRI offers superior assessment, it is less accessible due to cost, the need for specialized equipment, and expertise	Enhancing the availability of cardiac MRI and training more specialists, alongside using echocardiography when MRI is not feasible	[48,51,52]
Differentiation from other heart conditions	Obesity-related HFpEF can present similarly to other heart failure phenotypes, complicating differential diagnosis	Implementing a comprehensive diagnostic approach using a combination of clinical, biomarker, and imaging data to improve accuracy	Current study

Clinical management of obesity-related HFpEF

Managing obesity-related HFpEF requires a multifaceted approach that includes lifestyle modifications, pharmacological therapies, and management of comorbidities. Given the complex pathophysiology of the condition, treatment strategies should target underlying mechanisms to relieve symptoms and improve cardiovascular function.

Lifestyle Interventions

Weight loss through diet and exercise is central to managing obesity-related HFpEF. Even modest weight reduction can improve cardiac function, alleviate symptoms, and enhance quality of life [53,54]. Weight loss reduces volume overload, mitigates systemic inflammation, and improves diastolic dysfunction, the hallmark of HFpEF [55]. Additionally, it lowers blood pressure, enhances insulin sensitivity, and reduces the burden of comorbid conditions such as OSA, further benefiting cardiac function [56].

Exercise, mainly aerobic and resistance training, also plays a key role in improving cardiovascular health in obese HFpEF patients [57]. Regular physical activity enhances exercise tolerance, improves diastolic function, and reduces hospitalizations due to heart failure [58]. Exercise programs should be individualized, combining aerobic activities (e.g., walking, cycling) with strength training to improve muscle mass and metabolic function [57]. Furthermore, exercise improves endothelial function, reduces sympathetic nervous system activity, and modulates inflammatory pathways, contributing to better cardiac performance in HFpEF [59].

While lifestyle modifications are highly beneficial, long-term success depends on sustained changes in diet and activity. Patients should receive personalized support involving dietitians, physical therapists, and other healthcare professionals to optimize adherence to weight loss and exercise regimens [60,61].

Pharmacological Therapies

Pharmacological treatments for obesity-related HFpEF remain challenging, as no medications are currently approved specifically for this condition. However, certain drugs have shown promise in improving outcomes by addressing underlying mechanisms such as volume overload, fibrosis, and inflammation.

Sodium-glucose cotransporter 2 (SGLT2) inhibitors, such as empagliflozin and dapagliflozin, are emerging as effective therapies for HFpEF, both in obese and non-obese patients [62,63]. Initially developed for type 2 diabetes, these drugs can improve cardiovascular outcomes in heart failure by reducing hospitalizations and enhancing exercise capacity [62,63]. SGLT2 inhibitors promote natriuresis (sodium excretion), reducing fluid retention and improving heart failure symptoms [64]. Furthermore, they possess anti-inflammatory and antifibrotic properties, potentially mitigating myocardial remodeling associated with obesity and HFpEF [65].

Mineralocorticoid receptor antagonists (MRAs), such as spironolactone, have also been studied for HFpEF. MRAs block the effects of aldosterone, a hormone that promotes sodium retention and fibrosis in the myocardium [66]. By inhibiting aldosterone, MRAs may reduce myocardial fibrosis, improve diastolic function, and alleviate symptoms in obese HFpEF patients [66,67]. While MRAs are still under investigation, some studies suggest they may be beneficial, particularly in patients with elevated filling pressures [68].

Angiotensin receptor blockers (ARBs), such as losartan and candesartan, could also help manage obesity-related HFpEF. ARBs block the effects of angiotensin II, which promotes vasoconstriction, fluid retention, and myocardial fibrosis [69]. In HFpEF patients, ARBs can help reduce left ventricular filling pressures, improve myocardial relaxation, and lower the risk of hospitalization for heart failure [70]. However, evidence supporting the use of ARBs specifically for obesity-related HFpEF is still evolving, and more research is needed to determine their definitive role.

Despite the promising effects of these therapies, no single pharmacological treatment currently targets obesity-related HFpEF directly [71]. Ongoing research is crucial to identify more specific therapies that address the unique pathophysiology of this condition.

Management of Comorbidities

Effective management of comorbidities is crucial to improve outcomes in obese HFpEF patients, as conditions such as hypertension, diabetes, and OSA significantly influence the progression of the disease. Addressing these comorbidities can improve both symptoms and quality of life.

Hypertension is a common comorbidity in obesity-related HFpEF and a major contributor to diastolic dysfunction [37,38]. Blood pressure control is critical and should combine lifestyle modifications (e.g., dietary changes and weight loss) with pharmacological therapies, including ARBs, Angiotensin-converting enzyme inhibitors, and calcium channel blockers [72]. Adequate blood pressure management can reduce left ventricular filling pressures and improve diastolic function in obese HFpEF patients [73].

Diabetes and insulin resistance are also frequently associated with obesity-related HFpEF and contribute to myocardial injury and fibrosis. Tight glycemic control with medications such as SGLT2 inhibitors and metformin can reduce cardiovascular damage, which provides additional benefits for HFpEF management in obese patients [62-65].

OSA is another prevalent comorbidity in obesity, exacerbating the pathophysiology of HFpEF. OSA causes intermittent hypoxia, increased sympathetic tone, and systemic inflammation, all of which worsen diastolic dysfunction [39,40]. Treating OSA with continuous positive airway pressure therapy can improve sleep quality, reduce inflammation, and enhance cardiovascular function in obese HFpEF patients [74,75].

In addition to these conditions, regular screening for dyslipidemia and chronic kidney disease is crucial for comprehensive care [76]. Managing these comorbidities reduces the overall cardiovascular burden and improves the prognosis for obese HFpEF patients [77]. Table 2 summarizes key management strategies.

**Table 2:** Clinical management strategies for obesity-related HFpEF. HFpEF: heart failure with preserved ejection fraction; OSA: obstructive sleep apnea; CPAP: continuous positive airway pressure; ACEI: angiotensin-converting enzyme inhibitor; ARB: angiotensin receptor blocker; LV: left ventricle; SGLT2: sodium-glucose cotransporter 2; MRA: mineralocorticoid receptor antagonist

Component	Strategy	Rationale/Effectiveness	Reference
Lifestyle interventions	Weight loss	Even modest weight reduction improves cardiac function, reduces symptoms, and enhances quality of life	[53,54]
	Exercise (aerobic and resistance)	Improves cardiovascular health, enhances exercise tolerance, and reduces the risk of hospitalization	[57,58]
	Dietary modifications	Decreases volume overload, reduces systemic inflammation, and helps in blood pressure control	[55]
	Personalized support (dietitians, physical therapists)	Ensures sustained adherence to weight loss and exercise programs for long-term management	[60,61]
Pharmacological therapies	SGLT2 inhibitors (e.g., empagliflozin, dapagliflozin)	Reduces hospitalizations, improves exercise capacity, promotes natriuresis, and has anti-inflammatory and antifibrotic effects	[62-65]
	MRAs (e.g., spironolactone)	Reduces myocardial fibrosis, improves diastolic function, and can reduce symptoms, particularly in patients with elevated filling pressures	[66-68]
	ARBs (e.g., losartan, candesartan)	Reduces LV filling pressures, improves myocardial relaxation, and decreases hospitalization risk	[69,70]
Management of comorbidities	Hypertension control	Blood pressure management (via ARBs, ACE inhibitors, calcium channel blockers) reduces LV filling pressures and improves diastolic function	[72,73]
	Diabetes and insulin resistance management	Tight glycemic control with SGLT2 inhibitors or metformin improves glucose control and provides cardiovascular benefits	[62-65]
	OSA management (CPAP therapy)	Improves sleep quality, reduces systemic inflammation, and enhances cardiovascular function	[74,75]
	Other comorbidities (e.g., dyslipidemia, chronic kidney disease)	Regular screening and management help reduce the heart burden and improve prognosis in obese HFpEF patients	[76,77]

Emerging therapeutic approaches and future directions for obesity-related HFpEF

The treatment landscape for obesity-related HFpEF is rapidly evolving, with research increasingly focused on novel therapeutic targets, precision medicine, and expanded clinical trials. These efforts aim to refine disease management and develop tailored interventions that address the condition’s underlying pathophysiology. While current treatments offer some benefits, more targeted therapies are needed to improve outcomes in obese HFpEF patients.

Novel Therapeutic Targets

Emerging therapies for obesity-related HFpEF increasingly target key pathophysiological mechanisms, including inflammation, fibrosis, and metabolic dysregulation, each playing a crucial role in disease progression. Chronic low-grade inflammation, driven by adipose tissue, exacerbates systemic and myocardial inflammation, exacerbating diastolic dysfunction [78]. Targeting inflammatory pathways with monoclonal antibodies against pro-inflammatory cytokines, such as IL-6 and TNF-α, may help reduce myocardial inflammation and fibrosis [79]. Beyond cytokine inhibitors, therapies that modulate immune cell activity or signaling pathways could offer additional benefits [80]. However, the efficacy and safety of these approaches in obesity-related HFpEF remain under investigation [81].

Myocardial fibrosis, a hallmark of obesity-related HFpEF, contributes to increased ventricular stiffness and impaired diastolic function [82]. Novel antifibrotic strategies aim to reduce fibrosis by targeting the extracellular matrix or inhibiting the TGF-β pathway, which regulates collagen deposition [83,84]. Preclinical studies suggest that blocking fibrotic pathways can improve cardiac function [83], but large-scale clinical trials are needed to determine their effectiveness in obese HFpEF patients.

Metabolic dysfunction, including insulin resistance and impaired glucose metabolism, is a major contributor to obesity-related HFpEF [85]. Emerging therapies such as glucagon-like peptide-1 (GLP-1) receptor agonists and SGLT2 inhibitors show promise in improving both metabolic and cardiovascular outcomes [62,63,86]. These agents also reduce systemic inflammation, promote weight loss, and enhance myocardial function [64,65]. Further research is necessary to assess their long-term benefits in managing HFpEF in obese individuals.

Precision Medicine

The concept of precision medicine, which tailors treatment strategies based on individual patient characteristics, holds significant promise for managing obesity-related HFpEF. By integrating genetic, metabolic, and phenotypic data, personalized approaches can optimize outcomes by targeting the specific mechanisms driving HFpEF in each patient.

Genetic profiling may help identify gene variants that influence susceptibility to obesity, metabolic dysfunction, or myocardial remodeling, offering valuable insights for personalized treatment [87]. For instance, genetic markers linked to inflammation or fibrosis could help pinpoint patients who may benefit from targeted biologic therapies. Additionally, understanding the genetic underpinnings of obesity-related HFpEF could enable early identification of high-risk individuals, allowing for timely intervention [88].

Metabolic profiling is another critical component of precision medicine in HFpEF. Obesity-related metabolic dysfunction, including insulin resistance, altered lipid metabolism, and adipokine imbalances, varies widely among individuals [89]. Personalized treatments targeting specific metabolic abnormalities, such as SGLT2 inhibitors for insulin resistance or GLP-1 agonists for weight loss, may improve cardiac function and slow disease progression [62,63,86]. Tailoring therapy to a patient’s metabolic profile could enhance treatment efficacy and pave the way for more targeted interventions [90,91].

In addition, phenotypic data, including imaging studies and biomarkers, can help assess the extent of myocardial remodeling, fibrosis, and diastolic dysfunction in individual patients [92,93]. Correlating these characteristics with treatment response may allow clinicians to predict which therapies will be most effective, leading to more precise and personalized management of HFpEF [94].

Clinical Trials

The inclusion of obese patients with HFpEF in clinical trials is essential for developing evidence-based treatment guidelines specific to this phenotype. Historically, HFpEF trials have often excluded or underrepresented obese individuals, limiting the applicability of their findings to this high-risk population [95]. With obesity prevalence rising globally, there is an urgent need for dedicated clinical trials to evaluate the safety, efficacy, and long-term benefits of therapies tailored to obesity-related HFpEF [96].

These trials are crucial for assessing novel treatments targeting key obesity-related mechanisms, such as inflammation, fibrosis, and metabolic dysregulation. They should explore more therapeutic options, including pharmacological agents such as SGLT2 inhibitors and anti-inflammatory biologics, alongside lifestyle interventions such as exercise and dietary modifications. Determining the most effective strategies for improving outcomes in this population requires a comprehensive, evidence-based approach. Clinical trials should also incorporate biomarkers and imaging modalities to refine diagnostic and monitoring strategies. This helps optimize the use of existing diagnostic tools while ensuring that the benefits of emerging treatments are accurately measured. Multicenter collaborations and large-scale trials will be essential for generating robust data on safety and efficacy across diverse populations, ultimately guiding the development of targeted management strategies for obesity-related HFpEF. Table 3 outlines key emerging therapeutic strategies and future research directions.

**Table 3:** Emerging therapeutic strategies and research directions for obesity-related HFpEF. HFpEF: heart failure with preserved ejection fraction; SGLT2: sodium-glucose cotransporter 2; GPL-1: glucagon-like peptide-1; ECM: extracellular matrix; TGF-β: transforming growth factor-beta; TNF-α: tumor necrosis factor-alpha; IL-6: interleukin-6

Therapeutic area	Key strategies/Interventions	Research and considerations	References
Anti-inflammatory therapies	Monoclonal antibodies targeting pro-inflammatory cytokines (e.g., IL-6, TNF-α)	Aimed at reducing myocardial inflammation and fibrosis	[78-81]
		Efficacy and safety are still under investigation	
Fibrosis modulation	Antifibrotic agents targeting ECM and TGF-β pathway (collagen deposition)	Preclinical studies show potential for reducing myocardial fibrosis	[82-84]
		Large-scale clinical trials are needed for efficacy	
Metabolic dysregulation	Use of GLP-1 agonists and SGLT2 inhibitors	Promising for improving glucose control, reducing inflammation, promoting weight loss, and improving myocardial function	[62-65,85,86]
Genetic profiling	Identifying gene variants linked to obesity, metabolic dysfunction, and myocardial remodeling	Potential for personalized treatments targeting inflammation or fibrosis	[87,88]
		Early intervention for high-risk individuals	
Metabolic profiling	Tailored therapies based on individual metabolic abnormalities (e.g., SGLT2 inhibitors, GLP-1 agonists)	Varies between individuals, emphasizing precision medicine	[89-91]
		Potential for more targeted interventions	
Imaging and biomarkers	Use of imaging studies and biomarkers to assess myocardial remodeling, fibrosis, and diastolic dysfunction	Potential for predicting treatment response and improving individualized management	[92-94]
Clinical trials	Focused trials on obese HFpEF patients	Ensuring inclusion of obese individuals in clinical trials	[95,96]
	Testing pharmacological agents and lifestyle interventions (e.g., exercise, diet)	Need for large-scale, multicenter studies for robust data	

## Conclusions

Obesity-related HFpEF is a complicated condition driven by metabolic dysfunction, inflammation, myocardial remodeling, and comorbidities such as hypertension and diabetes. Current treatments, including lifestyle modifications and pharmacological therapies, provide limited benefits, underscoring the need for more targeted approaches. Emerging therapies, such as anti-inflammatory agents, antifibrotic drugs, and metabolic modulators such as SGLT2 inhibitors and GLP-1 receptor agonists, show promise in improving outcomes. Additionally, precision medicine, which tailors treatment based on genetic and metabolic profiles, may further enhance therapeutic effectiveness.

Future research must prioritize several critical areas: (1) large-scale clinical trials specifically including obese HFpEF patients to establish treatment efficacy; (2) development of obesity-specific diagnostic criteria to improve early detection; (3) investigation of novel biomarkers that better reflect disease severity in obesity; and (4) evaluation of combination therapies targeting multiple pathophysiological mechanisms simultaneously. Addressing obesity as a central factor in HFpEF progression will help refine diagnostic and management strategies. This integrated approach, combining lifestyle interventions, novel pharmacological treatments, and personalized medicine, represents an urgent clinical need that requires collaborative research efforts to ultimately improve outcomes for this growing patient population.
